# A Case Series of Severe Hyperammonemia Encephalopathy Related to Valproate: Can Antipsychotics Increase the Risk?

**Published:** 2019-07

**Authors:** Effat Davoudi-Monfared, Mojan Radmehr, Padideh Ghaeli, Maryam Mousavi

**Affiliations:** 1 Department of Clinical Pharmacy, School of Pharmacy, Tehran University of Medical Sciences, Tehran, Iran.; 2 Roozbeh Psychiatric Hospital, Tehran University of Medical Sciences, Tehran, Iran.; 3 Research Center for Rational Use of Drugs, Tehran University of Medical Sciences, Tehran, Iran; Roozbeh Hospital, Tehran University of Medical Sciences, Tehran, Iran.

**Keywords:** *Antipsychotic*, *Encephalopathy*, *Hyperammonemia*, *Valproate Derivatives*

## Abstract

Valproate-induced hyperammonemia is a common side effect of valproate, which may occur either without any symptoms or may rarely cause symptoms of encephalopathy. Different risk factors have been defined for this side effect, including some nutritional deficiencies and polypharmacy (eg, other anticonvulsants). Three cases with psychiatric disorder who showed symptoms of severe hyperammonemia encephalopathy and had taken valproate with antipsychotics, especially risperidone, are presented here. In all cases, the symptoms were improved by discontinuation of valproate. Administration of antipsychotic may be considered as a risk factor for hyperammonemic encephalopathy related to valproate, specifically in some prone populations.

Valproate-induced hyperammonemic encephalopathy (VHE) may occur in 16%-80% of patients receiving valproate. The range of incidence is wide due to variation in definition and setting. This adverse reaction can be either asymptomatic or presented with symptoms such as lethargy, drowsiness, and disorientation (1). Most of the reports have originated from neurology centers, but some recent reports have been received from psychiatric centers. Symptoms of VHE, such as lethargy, retarded motor symptom, or confusion may be misdiagnosed with exacerbation of current mood disorder (2). As a result, the doctor may increase the dose of valproate, which in turn can result in worsening the patient's condition. Hence, medical team awareness may prevent further complications. Various risk factors have been identified that correlate with VHE, which include age younger than two or over 65 years, taking multiple antiepileptics (especially phenytoin, phenobarbital, carbamazepine, and topiramate), developmental disability (such as autistic spectrum), vegetarian or vegan diets, urea cycle disorder, and hypercatabolic state (eg, burn and trauma). 

Also, some other medications may cause hyperammonemia encephalopathy and the exact relevance with valproate may not be possible. However, the correlation of VHE with some factors such as dose and serum medication level still remains unknown (3). Although the exact mortality of this side effect is not determined, some fatalities have been reported, which highlights the importance of early diagnosis and proper treatment of this adverse effect (4). In this article, three cases from Rouzbeh hospital, with symptoms of severe encephalopathy and extremely high serum ammonia levels due to consumption of valproate sodium and antipsychotics, were described.

***Case 1***

Z.G., a 35-year-old woman suffering from schizophrenia, was brought to the emergency ward because of her aggressive behavior and thoughts of suicide. At admission, the patient behaved aggressively, was agitated, paranoid and depressed, and felt guilty about how she had been mistreating her husband. She complained about her difficulty falling asleep. 

She also mentioned that she recently heard a voice commanding her to kill herself. Oral valproate 800 mg daily in divided doses and quetiapine 50 mg every night had been started for one week before admission. On the first day of admission, valproate was continued and quetiapine was changed to risperidone 2 mg daily in divided doses. Medication was continued for six days when the patient committed an unsuccessful suicide by cutting her wrist using sharp corners of a metal window. The symptoms of agitation and difficulty falling asleep were also observed. Thus, valproate and risperidone were increased to 1000 mg and 4 mg daily, respectively, and the valproate (VPA) level and liver enzyme were checked the next day. One week later, VPA level was reported to be 128 mg/L, with the normal range being 50-100 mg/L, and liver enzyme was found to stand within the normal limit.

Although death wish and auditory hallucination were improving, the nurses reported ataxia and delirious behavior at the ninth day of admission. Therefore, valproate was decreased to 750 mg daily and risperidone was increased to 6 mg daily, both in divided doses. Biperiden was also started at 3 mg daily in divided doses due to extrapyramidal effect induced by risperidone, and the patient was sent for electro-convulsive therapy (ECT). On the 10th day, the patient was delirious and disoriented to place and time. Therefore, valproate was discontinued. However, the patient was still suffering from delirium. She was sometimes unaware of the place and time and sometimes about both.

Carbamazepine 200 mg daily was started on day 17 and ECT was done every other day until day 20, when symptoms of delirium were exacerbated and carbamazepine, risperidone, biperiden, and ECT were all used by the patient. Haloperidol was started on 1 mg daily in divided doses the next day to help treat delirium. After day 20, haloperidol was continued, and the patient experienced severe psychomotor retardation 4 days later for which amantadine and biperiden were ordered. At the same time, serum ammonia was also checked due to the probability of encephalopathy. A few days later, the ammonia level was 297 (with a normal range of 18.7-86.9 mcg/dL) and L-carnitine 250 mg was started. One day later, the patient’s family insisted to discharge the patient from the hospital and she was discharged with legal self-consensus of her family. Other medications that were prescribed during her stay were propranolol, zinc sulfate, ferrous sulfate, folic acid, clonazepam, and acetaminophen. No significant abnormalities were found in the laboratory tests.

***Case 2***

G.T., a 54-year-old man, was admitted to the emergency ward due to paranoid delusion, aggressiveness, talkativeness, increased energy, and decreased need for sleep. He was a known case of bipolar disorder with a psychotic feature and had been admitted repeatedly in the same center during the past 30 years. The main etiology for his readmission was that his paranoid thoughts about his wife had returned. He believed that his wife was betraying him and that she intentionally was not giving him the medications to worsen his medical condition. He had also been diagnosed with multiple sclerosis (MS) since 2 years ago and was receiving glatiramer acetate 3 days a week. His medication history consisted of valproate sodium 1500 mg daily in divided doses and propranolol 10 mg thrice daily, which was stopped in the previous week, biperiden 2 mg twice daily that had been discontinued for at least 4 months and haloperidol with unknown order of prescription. Additionally, he was taking opium for several years and was on methadone and buprenorphine intermittently.

The patient was admitted because of exacerbation of the manic episode; however, it seemed that the underlying disease (MS) and poor adherence to medications caused him such an overwhelming anxiety that resulted in aggressiveness and aggravation of paranoid thoughts in return. The same medications with the same doses were reordered with the exception of haloperidol that was discontinued and replaced by olanzapine 10 mg in 2 divided doses. Also, lorazepam, biperiden, and clonidine (for drawal symptoms) were added to his medication. Due to excess sedation, it was decided to decrease the dose of sodium valproate on the third day, and as sedation continued, haloperidol was started on the seventh day and olanzapine was discontinued. Also, clonidine and buprenorphine use was limited. The patient started to feel better and the paranoid thoughts were disappearing. On the 14th day, the patient presented with a shuffling gate for which neurology consultation was requested. Neurology consultation was done and it was proposed that atypical antipsychotics were better options because of his underlying disease (MS) and probable drug-induced Parkinsonism. Once again, it was decided to replace haloperidol with olanzapine. Moreover, it was proposed that valproate could be discontinued because of excessive sedation. Hence, it was decided to taper down sodium valproate gradually because of mild abnormalities in electroencephalogram. On the 16th day, gait abnormalities, rigidity, and bradykinesia were not improved. At this time, serum valproate and ammonia levels were requested. On day 28, the ammonia level was reported to be 471 (with a normal range of 18.7-86.9 mcg/dL), the valproate level was reported as 94.5 mcg/mL and hepatic enzymes were within normal limits. Valproate was discontinued and L-carnitine 500 mg daily was started because of probable hyperammonemia encephalopathy. Four days later, the patient received methylprednisolone 500 mg for 5 days due to a new lesion on MRI. He had no problem during induction treatment of his MS attack. Other medications that he had received during admission were donepezil, amantadine, folic acid, vitamin B6, and gabapentin. Valproate was not started again for him and his mood was also stable until the next week when he was discharged and prescribed quetiapine 50 mg and carbamazepine 300 mg both in divided doses. Other medications were calcium supplement and folic acid and he was told to visit his neurologist to decide for a proper treatment for his underlying disease. One month later, he came back for a visit and showed no sign of aggressiveness and paranoid thoughts. 

***Case 3***

S.Y was a 49-year-old woman who was a known case of bipolar disorder for 25 years. She was admitted to the emergency ward because of her aggressive symptoms and tendency to harm herself and others. She was aggressive, agitated, and paranoid about her mother and sister; she was also depressed and had thoughts of suicide and self-injury. She also complained about insomnia and staying awake most of the nights. She was taking valproate 500 mg 3 times a day and risperidone 2 mg at bedtime for many years. On the first day of admission, her valproate was continued and risperidone dose was increased to 1.5 mg twice daily, and on the second day, her risperidone was increased to 2 mg twice daily. Four days later, she lost consciousness and her GCS reduced to 7-8. On day 6, her consciousness did not improve and VPA level was requested. Brain CT scan was performed and there were no signs of end-organ damage. All vital signs were normal except blood pressure that stood above 150/110 mmHg. In the evening of the sixth day of admission, she became conscious but disoriented to time, place, and people. On day 7, she was unconscious again and did not show any responses to painful stimuli. Her VPA level was 201 mcg/mL. Meanwhile, ammonia level was also examined. Her VPA was discontinued and lithium was started. L-carnitine 1000 mg 3 times a day was also started. Risperidone was stopped and replaced by haloperidol 5 mg daily. On day 9, she became alert and oriented. A few days later, her ammonia level was reported at 327mcg/dL. Her hepatic enzyme and other laboratory tests were all within normal limit. Her therapy was continued with lithium and haloperidol; and during her stay in the hospital, she did not experience further loss of consciousness and disorientation. She was discharged and was taking lithium, quetiapine, trifluoperazine, biperiden, and propranolol. She came for a visit 2 weeks later and her only complaint was fatigue, and her other aggressive and agitated symptoms were gone.

  The name of hospital is hidden because of regulation of submission to journal

## Discussion

VHE is a serious adverse effect of valproate that can even lead to death but can be prevented by precautious diagnosis and lowering the dose or discontinuing the medication. Different risk factors may be related to this adverse effect, e.g., urea cycle disorder, immature hepatic function, hereditary or dietary-induced carnitine deficiency, comorbid diseases, increased protein load, and polypharmacy (5). VHE mostly coincides with normal hepatic function (6).

The mechanism of VHE is related to both renal and hepatic metabolic pathways (7).The renal pathway that has a minor role is related to stimulation of renal glutaminase by one of the metabolites of valproate, sodium 2-propyl 4-pentenoate (4-en-VPA). This may increase renal glutamine uptake and ammonia release [8]. Additionally, the hepatic pathway has a major and complex role in increased ammonia level. One arm is inhibition of the enzyme carbamoyl–phosphate synthetase I (CPS-I) that leads to an increase in ammonia level. Another arm is an increase in omega-oxidation pathway of valproate metabolism to produce 4-en-VPA and propionate that causes hyperammonemia. Propionate also decreases hepatic N-acetyl glutamate level and causes an increase in ammonia level due to CPS-I inhibition. Carnitine deficiency may result in valproate toxicity, especially in long-term.

The symptoms of VHE vary, although it can also be asymptomatic. VHE can be seen in the form of acute onset of lethargy, impaired consciousness, disorientation, cognitive slowing, and focal neurological deficits (3). The symptoms of the patient in case 1 was disorientation and slowing of cognition; case 2 showed excess sedation and impaired cognition. Both cases showed some degree of extrapyramidal side effects, such as shuffling or bradykinesia. Symptoms of case 3 consisted of sudden loss of consciousness. However, all three cases showed improvement in their symptoms after discontinuation of valproate.

In fact, VHE is very rare when valproate is used alone and mostly occurs in patients taking valproate with other anticonvulsant drugs, such as carbamazepine or phenytoin (9). Beside anticonvulsants, some other psychotropics, such as risperidone, have been reported to induce VHE (10, 11). 

Several cases have been reported about risperidone-related VHE and the interaction between risperidone and valproate. Carlson et al reported two pediatric cases with valproate-induced hyperammonemia related to risperidone and suggested that some genetic predisposition (for example, unknown mitochondrial dysregulation) may exist that make children prone to this adverse effect (10).

Another theory for this interaction may contribute to the ability of risperidone to inhibit CYP2D6 (albeit weekly), and it was proposed that risperidone can increase VPA level (12). Sund et al showed that this may not be true, as in their study, there was no difference between the serum level of valproate in patients who take risperidone and those who do not (13).Another more acceptable theory, although not fully examined, is that valproate extensively binds to plasma proteins. Risperidone may compete with valproate in binding to proteins and may increase the unbound drug in blood, which may lead to further toxicity (10).

The other mechanism that can lead to risperidone-valproate interaction and valproate toxicity may be related to CYP2D6 polymorphism. One of the most prominent pathways of metabolizing risperidone is CYP2D6, and some studies have shown that CYP2D6 poor metabolizers may have higher risk of side effects (14).Studies have also shown that Caucasians have high poor metabolizer ratios of CYP2D6 (15). It can be assumed that from the overall population, poor metabolizers are at the unpredictable risk of interaction between valproate and risperidone due to the longer half-life of risperidone. 

One concerning aspect of VHE is that it can happen anytime during the treatment, even after years (16). Another concern is misdiagnosis of this side effect with other mood disorder and adverse effects. Likewise, in this study, case two and three have been taking valproate for years. Also, their symptoms were not initially assumed as VHE symptoms. Hence, it may be necessary to request an ammonia level under special conditions or determined interval in prone patients. However, being prone to this side effect should be clarified more precisely in future investigations to prevent overdiagnosis. 

Based on the authors' experiences, the incidence of severe hyperammonemia increases when valproate is used with antipsychotics, especially risperidone, in Iranian population. The reason this was not reported before might be attributed to either the limitation in laboratory testing or the lack of proper diagnosis of this side effect. The authors are planning to design new studies to investigate other risk factors for VHE in our ethnic group. 

In these cases, G.T. was diagnosed with hyperammonemia encephalopathy irrespective of risperidone, but it may contribute to olanzapine or haloperidol. Olanzapine administration has never been assumed as a risk factor for VHE; there are some reports of VHE that patients have taken olanzapine with valproate (17, 18), although the exact correlation of VHE and coadministration of olanzapine is not fully explained. Which antipsychotics may be more prominent risk factors for VHE is a question that can be answered in future investigations.

## Conclusion

Hyperammonemic encephalopathy is a potentially serious side effect of treatment with valproate. Different risk factors, such as age, urea cycle disorder, concomitant administration of some antiepileptic, and nutritional deficiency have been proposed for incidence of VHE. In this case series, 3 cases of severe VHE that may be contributed to coadministration of antipsychotics, especially risperidone, were described. The symptoms of all three cases were improved after discontinuation of valproate. However, this was a hypothesis and needs larger studies to clarify the exact contributors and risk factors for VHE, especially in Iranian populations.
